# Neutral Effect of Skeletal Muscle Mineralocorticoid Receptor on Glucose Metabolism in Mice

**DOI:** 10.3390/ijms24087412

**Published:** 2023-04-18

**Authors:** Alessandra Feraco, Stefania Gorini, Caterina Mammi, Mauro Lombardo, Andrea Armani, Massimiliano Caprio

**Affiliations:** 1 Department of Human Sciences and Promotion of the Quality of Life, San Raffaele Roma Open University, 00166 Rome, Italy; alessandra.feraco@uniroma5.it (A.F.);; 2 Laboratory of Cardiovascular Endocrinology, IRCCS San Raffaele, 00166 Rome, Italy

**Keywords:** insulin resistance, insulin signaling, obesity, lipid infiltration, inflammation, myokines

## Abstract

The mineralocorticoid receptor (MR) is able to regulate the transcription of a number of genes in the myotube, although its roles in skeletal muscle (SM) metabolism still await demonstration. SM represents a major site for glucose uptake, and its metabolic derangements play a pivotal role in the development of insulin resistance (IR). The aim of this study was to investigate the contribution of SM MR in mediating derangements of glucose metabolism in a mouse model of diet-induced obesity. We observed that mice fed a high-fat diet (HFD mice) showed impaired glucose tolerance compared to mice fed a normal diet (ND mice). Mice fed a 60% HFD treated with the MR antagonist Spironolactone (HFD + Spiro) for 12 weeks revealed an improvement in glucose tolerance, as measured with an intraperitoneal glucose tolerance test, compared with HFD mice. To investigate if blockade of SM MR could contribute to the favorable metabolic effects observed with pharmacological MR antagonism, we analyzed MR expression in the gastrocnemius, showing that SM MR protein abundance is downregulated by HFD compared to ND mice and that pharmacological treatment with Spiro was able to partially revert this effect in HFD + Spiro mice. Differently from what we have observed in adipose tissue, where HDF increased adipocyte MR expression, SM MR protein was down-regulated in our experimental model, suggesting a completely different role of SM MR in the regulation of glucose metabolism. To confirm this hypothesis, we investigated the effects of MR blockade on insulin signaling in a cellular model of IRin C2C12 myocytes, which were treated with or without Spiro. We confirmed MR protein downregulation in insulin-resistant myotubes. We also analyzed Akt phosphorylation upon insulin stimulation, and we did not observe any difference between palmitate- and palmitate + Spiro-treated cells. These results were confirmed by in vitro glucose uptake analysis. Taken together, our data indicate that reduced activity of SM MR does not improve insulin signaling in mouse skeletal myocytes and does not contribute to the favorable metabolic effects on glucose tolerance and IR induced by systemic pharmacological MR blockade.

## 1. Introduction

Mineralocorticoid receptor (MR) activation by aldosterone plays a crucial role in the distal nephron in the regulation of sodium levels and blood pressure [1,2]. Besides this well-known function in the kidney, the MR is expressed also in the vasculature and adipose tissue (AT), and its altered function in these tissues favors vascular and metabolic dysfunctions [3]. High plasma levels of aldosterone have been detected in subjects with obesity and metabolic syndrome (MetS), suggesting a positive association between aldosterone levels, MR activation, and cardiometabolic risk [4].

Hyperactivation of vascular MR has deleterious effects, resulting in increased oxidative stress, which in turn leads to reduced NO production and favors endothelial dysfunction [5,6,7,8]. Preclinical studies also reveal that increased plasma levels of aldosterone stimulate atherosclerosis development [9,10,11,12]. On the other hand, in obese mice that showed vasoconstrictor response and endothelial dysfunction, mineralocorticoid receptor antagonist (MRA) treatment led to reduced expression of pro-oxidative NADPH oxidase and increased levels of the antioxidative enzymes glutathione peroxidase-1 and superoxide dismutase-1 and -3 in endothelial cells, with potential protection against endothelial alterations [5], suggesting that MRA administration may represent a valid therapeutic strategy to counteract vascular dysfunction and atherosclerotic complications induced by MR overactivation. Accordingly, ApoE KO mice treated with MRA show reduced atherosclerotic lesion size [13].

Preclinical studies have shown that mice with overexpression of adipocyte-specific MR show increased local oxidative stress and altered vascular contractility [14,15,16]. As regards the impact of MR on metabolic dysfunction, it has been established that MR gene expression is increased in visceral adipose tissue in a preclinical mouse model of MetS as well as in obese patients. In particular, conditional MR overexpression in the adipocyte led to increased body weight, fat mass expansion, and the onset of insulin resistance (IR) and MetS in mice even in the absence of an obesogenic diet [17]. Accordingly, obese mice treated with MRA display reduced fat accumulation, decreased oxidative stress, and inflammatory cytokine expression, with a parallel improvement in insulin resistance (IR) [18,19]. In addition, MRA-treated obese mice show browning of white adipose tissue, possibly contributing to reducing weight gain and white fat mass and improving glucose metabolism [20]. In clinical studies, in patients with primary aldosteronism, MRA treatment has been shown to counteract IR and visceral fat mass expansion [3]. Administration of the MRA Spironolactone (Spiro) was also able to activate human brown adipose tissue (BAT), with potentially protective effects against obesity and altered glycemia control [21].

MR is expressed and functional in skeletal muscle (SM) fibers, and it has been established that MRAs improve SM pathology in Duchenne muscular dystrophy (DMD) mouse models [22]. Importantly, SM represents a major site for glucose uptake and storage, thus contributing to whole-body energy metabolism. Therefore, its metabolic derangements play a pivotal role in the development of IR and type 2 diabetes (T2D) [23]. Recently, it has been demonstrated that spironolactone administration inhibited western diet-induced glucose intolerance, and IR, and impaired insulin metabolic signaling in the soleus, which is rich in oxidative fibers, in mice. The properties of muscle fibers, i.e., their type, are important determinants of the functional characteristics of SM. Moreover, several studies suggest that insulin-mediated glucose metabolism is different between muscle fiber types [24,25,26]. The aim of our study was to investigate the role of MR in mediating metabolic alterations in the gastrocnemius (GA), which is mainly composed of glycolytic fibers, in a mouse model of diet-induced obesity.

## 2. Results

### 2.1. In Mice Fed a 60% HFD Spironolactone Significantly Improves Glucose Intolerance

**Figure 1:**
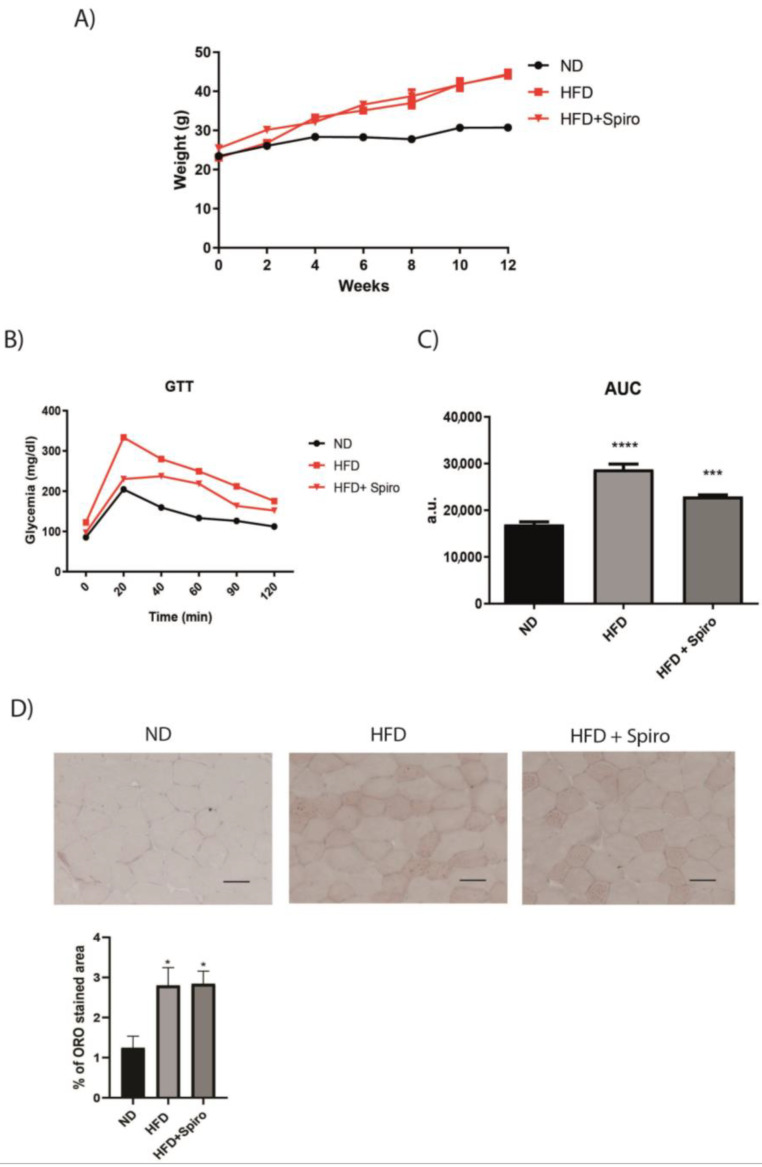
Spironolactone prevents impaired glucose intolerance without affecting body weight gain in mice fed a high-fat diet. (**A**) Body weight over the course of the study (n = 10). (**B**), Plasma glucose levels during the intraperitoneal glucose tolerance test (IPGTT) following food withdrawal for 6 h (n = 10). (**C**), The area under the curve (AUC) of glucose using the trapezoidal rule (n = 10). (**D**), Oil Red O staining on tibialis anterior. Representative sections from the tibialis anterior of all groups (n = 6, scale bar 50 µm). * *p* < 0.05 vs. ND; *** *p* < 0.001 vs. HFD; **** *p* < 0.0001 vs. ND. Values are expressed as means ± SEM.

In the present study, 10-week-old C57BL/6J male mice were fed a high fat diet (HFD) (60% calories from fat) for 12 weeks (HFD group), resulting in a significant increase in body weight compared with control mice (Figure 1A). Mice fed an HFD containing Spiro, corresponding to the HFD + Spiro group, showed a body weight similar to that of the HFD group (Figure 1A). The intraperitoneal glucose tolerance test (IGTT), performed after 12 weeks of diet, showed a significant increase in plasma glucose levels in the HFD group at 20 min after intraperitoneal glucose injection compared with the ND group (Figure 1B), leading to a significant increase in the area under the curve (AUC) of plasma glucose (Figure 1C). As expected and in accordance with previous papers [20], MR antagonism with Spiro prevented the HFD-induced increase in blood glucose after IPGTT, as shown by the significant reduction of the total glucose AUC (Figure 1C). In particular, at 20 min after intraperitoneal glucose injection, the HFD + Spiro group showed a significant reduction in plasma glucose levels compared with the HFD group (Figure 1B), indicating that Spiro was able to counteract HFD-induced glucose intolerance. A previous study by our group has shown that mice fed an HFD (60% calories from fat) and treated with MR antagonist Finerenone revealed BAT activation with concomitant improvements in glucose profiles. This study demonstrated that systemic MR blockade protects against BAT dysfunction and improves metabolic disorders associated with adipose tissue dysfunction [27]. In order to evaluate whether Spiro was able to protect from SM dysfunction induced by ectopic fat deposition in obesity, we investigated fat infiltration in the tibialis anterior (TA) isolated from HFD mice. We observed that Spiro treatment did not induce morphological changes in TA, since morphological analysis did not reveal a reduction in intramyocellular lipid accumulation between the HFD group and the HFD + Spiro group (Figure 1D).

### 2.2. MR Protein Expression Is Downregulated in SMin Obesity

**Figure 2:**
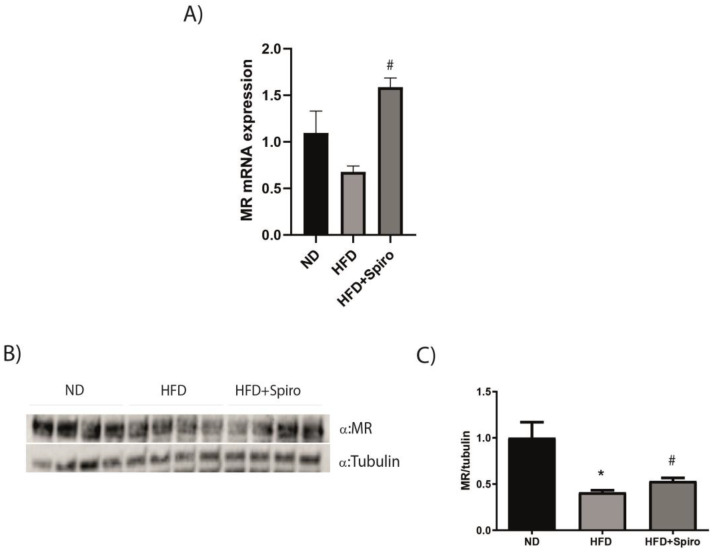
MR expression is downregulated by HFD compared to ND mice and Spiro treatment partially reverts this effect in HFD + Spiro mice. (**A**), qRT-PCR analysis of MR gene expression in GA of ND, HFD, and HFD + Spiro groups (n = 10). (**B**), representative immunoblots of MR expression analysis in GA (n = 10), and (**C**) distribution graphs of the densitometric scanning analyses performed by ImageQuant TL software by using α-tubulin as a loading control. * *p* < 0.05 vs. ND; # *p* < 0.05 vs. HFD. Values are expressed as means ± SEM.

MR expression is increased in adipose tissue in obesity, both in rodents and in humans [17]. We investigated whether the SM MR expression profile was similar to what was observed in the adipocyte. The analysis of MR gene expression in the GA from the HFD group revealed a significant downregulation in *MR* mRNA levels compared to ND mice (Figure 2A). Such a result was confirmed by Western blot analysis, which revealed that MR protein expression is downregulated by HFD compared to ND mice in GA (Figure 2B,C). SM MR downregulation did not counteract glucose intolerance induced by HFD in vivo (Figure 1B,C), suggesting that MR does not affect glucose metabolism in SM with obesity. Interestingly, Spiro treatment was able to partially revert this effect in HFD + Spiro mice (Figure 2A–C), indicating that systemic MR blockade is able to modulate MR expression in SM.

### 2.3. Spironolactone Does Not Improve Insulin Sensitivity in Obese Mice

**Figure 3:**
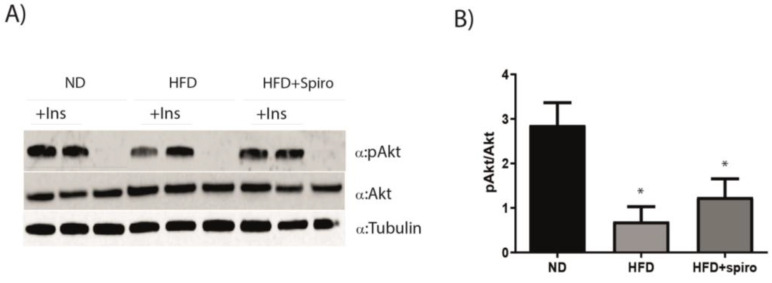
Mice fed an HFD displayed altered insulin signaling, but Spiro treatment does not improve insulin sensitivity in obese mice. (**A**), representative immunoblots of Akt activation analysis in GA (n = 10). (**B**), distribution graphs of the densitometric scanning analyses performed by ImageQuant TL software by using α-tubulin as loading control. Akt’s phosphorylated form was normalized in comparison with its total form. * *p* < 0.05 vs. ND. Values are expressed as means ± SEM.

In order to investigate whether MR blockade was able to influence insulin signaling in SM, preserving insulin sensitivity in this tissue in obesity, we treated mice with insulin or a vehicle for 15 min in experimental mice after 12 weeks on HFD or HFD plus Spiro treatment, and we measured Akt phosphorylation in the GA. Mice fed a HFD and injected with insulin displayed altered insulin signaling, as revealed by a significant reduction in insulin-induced pAkt protein expression in this group compared to controls. Nevertheless, systemic treatment with Spiro did not alter Akt phosphorylation in the HFD + Spiro group compared to HFD-fed mice, indicating that pharmacological MRA does not provide protection against SM IR in obese mice (Figure 3).

### 2.4. MR Antagonism Does Not Improve Insulin Sensitivity in a Model of Insulin Resistance in Cultured Murine Myocytes

**Figure 4:**
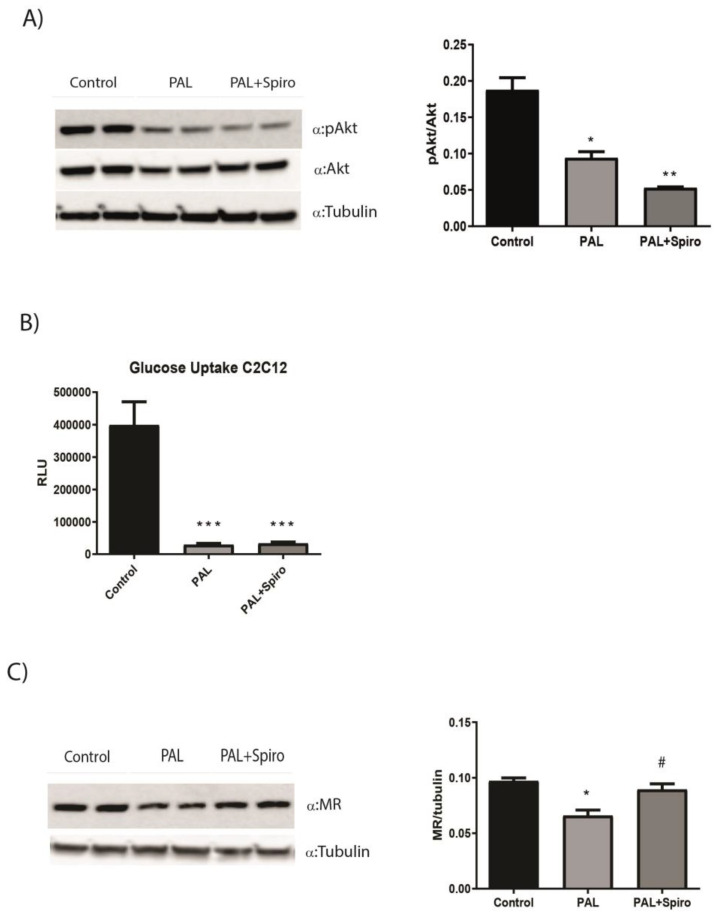
Spiro treatment does not improve insulin sensitivity in a model of insulin resistance in cultured murine myocytes (**A**), (**left panel**): representative immunoblots from 3 independent experiments for analysis of Akt activation in C2C12 myotubes treated with BSA/palmitate 0.75 mM and Spiro for 16 h and then stimulated with 100nM insulin for 15 min. (**Right panel**): distribution graphs of the densitometric scanning analyses performed by ImageQuant TL software by using α-tubulin as loading control. The phosphorylated form was normalized in comparison with its total form. (**B**), effect of Spiro on glucose uptake in C2C12 myotubes. Differentiated C2C12 myotubes were treated with BSA/palmitate 0.75 mM for 16 h in order to induce insulin resistance, in the presence or absence of Spiro. Glucose uptake in cells was determined using the Glucose Uptake-Glo Assay Kit (Promega). (**C**), representative immunoblots from 3 independent experiments for analysis of MR expression in C2C12 myotubes treated with BSA/palmitate 0.75 mM and Spiro for 16 h. (**Right panel**): distribution graphs of the densitometric scanning analyses performed by ImageQuant TL software by using α-tubulin as loading control. * *p* < 0.05 vs. control; ** *p* < 0.01 vs. control; *** *p* < 0.001 vs. control; # *p* < 0.05 vs. control. Values are expressed as means ± SEM.

In order to test Spiro’s impact on insulin signaling in vitro, we treated murine myotubes C2C12 with palmitate for 16 h in order to induce IR, in the presence or absence of Spiro. C2C12 myotubes cultured with palmitate displayed a significant reduction in pAkt protein expression compared to untreated controls. Spiro did not increase Akt phosphorylation in palmitate-treated cells (Figure 4A). Moreover, a glucose uptake assay performed in the same experimental study showed significant inhibition of cellular glucose uptake in C2C12 treated with palmitate (Figure 4B). In accordance with protein results, Spiro did not improve glucose uptake in myotubes with IR (Figure 4B), confirming that MR antagonism does not affect insulin signaling in SM in vitro. Of note, myotubes treated with palmitate showed a significant reduction in terms of MR protein expression compared with untreated cells (Figure 4C), thus confirming that IR induced by saturated fatty acids determines a downregulation of MR expression in SM in vitro, similar to what was observed in obese mice. Again, Spiro partially restored MR protein expression in C12C12 myotubes (Figure 4C).

### 2.5. MR Blockade with Spironolactone Modulates Myokine Profile in Obese Mice

**Figure 5:**
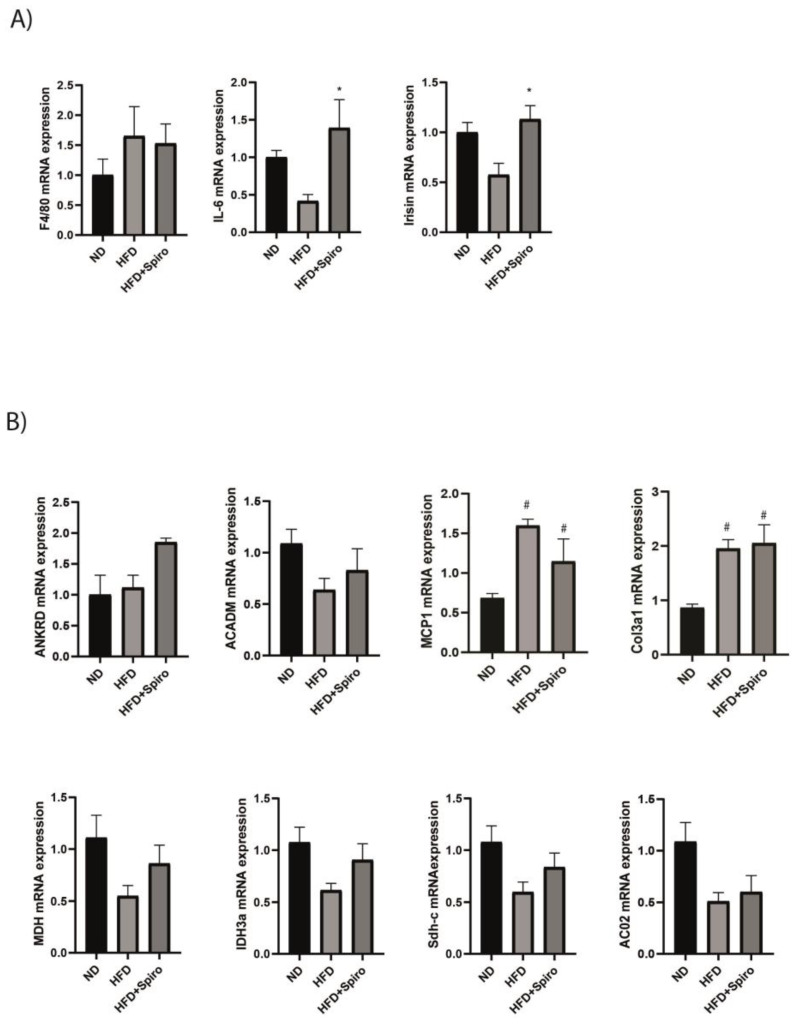
Specific MR blockade by spironolactone regulates the myokines profile of mice fed an HFD. (**A**), qRT-PCR analysis of genes related to F4/80, IL-6, and Irisin; (**B**), ANKRD, mitochondrial fatty acid uptake, inflammation, fibrosis, fatty acid oxidation (FAO), and the tricarboxylic acid cycle (TCA) in GA of ND, HFD, and HFD + Spiro groups (n = 10). * *p* < 0.05 vs. HFD; # *p* < 0.05 vs. ND. Values are expressed as means ± SEM.

In order to investigate whether MR modulation could influence different functions in SM, we analyzed gastrocnemius transcripts by qPCR. In accordance with histological results, macrophage marker *F4/80* expression, a well-known mediator of the diabetogenic properties of fatty acids, was not affected by Spiro (Figure 5A), confirming that MR blockade does not protect against macrophage infiltration induced by obesity. In addition, in our experimental model, HFD induced an increase in transcript levels of *MCP-1*, which represents a chemokine involved in monocyte recruitment [28]. The MR blockade did not blunt the inflammatory response in terms of *MCP-1* gene expression in the HFD + Spiro group (Figure 5B). On the other hand, *IL-6*, a myokine secreted in response to prolonged exercise and which acts as an energy sensor, was significantly upregulated by Spiro in the HFD + Spiro group compared with HFD-fed mice (Figure 5A). Similarly, the expression of *Irisin*, another myokine released into the circulation in response to exercise and involved in thermoregulation, was increased in the HFD + Spiro group compared with the HFD group (Figure 5A). IL-6 contributes to hepatic glucose production and enhances fat oxidation and lipolysis in SM during exercise [29,30,31]. We investigated whether Spiro influenced the expression of genes related to fatty acid oxidation (FAO), such as *ACADM*, and the tricarboxylic acid cycle (TCA), in the GA of HFD and HFD + Spiro. In the presence of Spiro, no difference was observed in gene expression of the TCA cycle or FAO markers. (Figure 5B, genes listed in Table 1). In order to investigate the contribution of MR to mitochondrial biogenesis, we analyzed the protein expression of the transcriptional regulator PGC-1α in GA. As expected, mice fed an HFD showed a significant reduction in PGC-1α protein levels. The MR blockade did not affect PGC-1α expression in the HFD + Spiro group. Accordingly, HFD-induced PGC-1α downregulation led to reduced expression of *mitochondrial transcription factor A (mtTFA)* as well as the nuclear-encoded mitochondrial subunit of the electron transport chain complex, *cytochrome c*. Spiro did not affect the mentioned mitochondrial biogenesis marker expression in mice challenged with HFD (Figure S1, genes listed in Table 1).

It is well established that obesity-associated metabolic alterations, such as IR and T2D, contribute to fibrosis development in SM [32]. For this reason, we investigated the expression of the extracellular matrix (ECM) remodeling marker *Col3a1* in GA, observing that Spiro did not blunt the upregulated expression of this marker in mice fed an HFD (Figure 5B).

Finally, Chadwick and colleagues demonstrated that MR antagonism by spironolactone was able to downregulate the *Ankrd1* transcript in a Duchenne muscular dystrophy (DMD) mouse model, thus identifying *Ankrd1* as a novel MR gene target [22]. By contrast, in our model, Spiro did not induce significant changes in *Ankrd1* gene expression, despite a positive trend in terms of increased mRNA levels observed in the HFD + Spiro group compared with HFD-fed mice (Figure 5B).

## 3. Discussion

We previously demonstrated that pharmacological MR blockade is protective against adipose tissue and glucose metabolism dysfunctions in obese mice [20,27]. SM is the primary site for glucose uptake and storage and has a major contribution to whole-body energy metabolism; therefore, its metabolic derangements play a pivotal role in the development of IR in obesity. Moreover, muscles serve as an important thermogenic organ [23,33,34]. In 2015, Chadwick and colleagues demonstrated for the first time that MR is expressed and functional in SM fibers and that MR antagonism can lead to beneficial gene expression changes in Duchenne muscular dystrophy (DMD) mouse models [22]. In this study, we investigated the contribution of MR to mediating SM metabolic alterations in a model of diet-induced obesity. Mice fed an HFD and treated with the MRA Spiro for 12 weeks revealed an improvement in glucose tolerance compared with HFD mice (Figure 1). We first characterized the expression of MR in GA in mice fed an HFD, and we observed a significant downregulation of MR protein as well as mRNA expression (Figure 2A). Such a pattern of expression is opposite to that observed in adipose tissue, where an obesogenic diet induced an increase in the expression of MR [17], thus suggesting that MR plays a different role in SM compared to adipose tissue. Moreover, in our experimental study, Spiro was able to raise MR protein expression almost to basal levels (Figure 2B,C), indicating that systemic MR blockade controls SMMR expression. It is well established that obesity promotes chronic low-grade inflammation due to the release of inflammatory factors and free fatty acids from adipose tissue, which alter the insulin signaling pathway in muscle cells [35,36]. In order to investigate the effect of Spiro on the insulin sensitivity of skeletal muscle, we treated experimental mice, after 12 weeks of HFD or HFD plus Spiro treatment, with insulin or vehicle for 15 min, and we analyzed Akt phosphorylation in GA. As expected, HFD led to impairment of the Akt pathway in SM; MR antagonism was not able to restore insulin-mediated phosphorylation of Akt in our model (Figure 3), indicating that MR downregulation or pharmacological MR blockade in SM are associated with impaired insulin signaling in this tissue. These data suggest that Spiro-induced improvement in systemic glucose tolerance is not mediated by local changes in insulin signaling in SM, excluding a role for muscle MR blockade in the protective effects of Spiro on glucose metabolism.

In the present study, we have investigated SM insulin signaling and glucose uptake in the presence of downregulated local expression or activity of MR. We did not investigate the metabolic effects of MR activity upregulation on SM glucose metabolism, and the lack of a transgenic mouse model that over-expresses MR in skeletal muscle may represent a limit for our study. However, it is unlikely that up-regulated local activity of MR exerts protective effects on glucose metabolism, whereas all studies on the up-regulated activity of MR show detrimental cardiometabolic effects [3].

To further investigate the role of MR on insulin signaling in muscle cells, we performed in vitro experiments using the most abundant dietary saturated fatty acid, palmitate, to induce IR in murine myocytes C2C12, as previously described [37]. Similarly to our findings in vivo, myotubes exposed to palmitate for 16 h displayed impairment in the Akt pathway compared to untreated controls, and MR antagonism was not able to restore Akt phosphorylation in palmitate-treated cells (Figure 4A). Notably, the glucose uptake assay, performed in the same experimental study, showed significant inhibition of glucose uptake in myocytes in the presence of palmitate (Figure 4B). In accordance with the Akt protein results, Spiro did not improve glucose uptake in myotubes with IR (Figure 4B), confirming that MR antagonism does not affect insulin signaling in SM in vitro. Of note, myotubes treated with palmitate showed a significant reduction in terms of MR protein expression compared with untreated cells (Figure 4C), thus confirming that IR is associated with downregulated MR expression in SM cells also in vitro, as observed in obese mice. Again, Spiro partially restored MR protein expression in C12C12 myotubes (Figure 4C). Importantly, in accordance with our data, a recent preclinical study provided evidence that a loss of MR signaling inhibits insulin-stimulated glucose uptake in the skeletal muscle, thus leading to altered SM metabolism. This study suggests that MR function is essential to maintain glucose uptake in muscle [38]. On the other hand, it has been shown that Spiro administration counteracted western diet-induced glucose intolerance, IR, and impaired insulin metabolic signaling in the soleus in mice [39]. Our findings about the effect of MR antagonism on insulin signaling in SM are in contrast with the above-mentioned study. This discrepancy might derive from different insulin-mediated glucose metabolism depending on muscle fiber types. Indeed, SM is a heterogeneous tissue composed of different fiber types, which can be distinguished according to myosin heavy chain (MHC) isoform expression. Soleus is composed of oxidative, fatigue-resistant, slow-twitch type I fibers, whereas the proportion of glycolytic, fast-twitch type II fibers is higher in GA [24,25,26]. Of note, reduced oxidative enzyme activity and increased glycolytic to oxidative enzyme activity have been found in obesity and type 2 diabetes [40,41], and several studies suggest that the proportion of type I fibers is lower compared with type II fibers in the presence of IR [42,43,44,45]. These data address the importance of considering muscle fiber types in terms of an appropriate model to explore specific metabolic aspects, such as insulin responses in vivo.

SM is now recognized as an endocrine organ, secreting several myokines that play a pivotal role in the cross-talk between SM and different organs, including insulin-target organs such as white and brown adipose tissue and the liver [46]. Interestingly, altered secretion of several myokines has been shown to be a hallmark of SM in insulin resistance [47]. In our study, GA transcript analysis revealed an effect of Spiro on the myokine profile. SM secretes a variety of proinflammatory molecules due to immune cell infiltration in obesity [48]. Indeed, macrophage markers are increased in the SM of type 2 diabetic patients and correlate with HOMA-IR [28]. F4/80 is a well-known mediator of the diabetogenic properties of fatty acids, and F4/80-positive macrophages have been found to be significantly increased in the GA of ob/ob mice compared to lean controls [28]. In our study, according to histological results, macrophage marker *F4/80* expression was not affected by Spiro (Figure 5A), indicating that MR blockade does not protect from immune cell infiltration induced by obesity. In addition, elevated circulating levels of MCP-1 are associated with SM inflammation [28] and positively correlate with fat mass expansion in obesity. Indeed, mice overexpressing MCP-1 in adipose tissue show IR features. Furthermore, it has been shown that MCP-1 treatment inhibits insulin-stimulated glucose uptake in myotubes ex vivo [49]. In our experimental model, *MCP-1* mRNA expression was upregulated by HFD. Nevertheless, Spiro did not significantly blunt the inflammatory response or macrophage infiltration in the HFD + Spiro group, as revealed by *F4/80* gene expression analysis (Figure 5A,B). On the other hand, *IL-6* was significantly upregulated by Spiro in the HFD + Spiro group compared with HFD-fed mice (Figure 5A). Interestingly, despite the well-described pro-inflammatory effects of IL-6 in metabolic disease, it has been shown that IL-6 released from SM in response to exercise exerts different effects, mostly acting in a hormone-like fashion. Indeed, the biological role of IL-6 is not limited to improving the metabolic profile but involves muscle growth, differentiation of satellite cells, browning of fat depots, and cardiovascular protection [50,51]. Similarly, the expression of *Irisin*, one of the most recently described myokines [52], released into the circulation in response to exercise and involved in thermoregulation, was increased in the HFD + Spiro group compared with the HFD group (Figure 5A). These results point out the complexity of the SM as an endocrine organ and the potential role of MR in SM physiology beyond metabolic homeostasis regulation. Notably, IL-6 contributes to hepatic glucose production and enhances fat oxidation and lipolysis in SM during exercise [31,53,54]. We investigated whether Spiro influenced the expression of genes related to fatty acid oxidation (FAO), such as *ACADM* and to the tricarboxylic acid cycle (TCA), in the GA of HFD and HFD + Spiro. In our model, Spiro did not affect gene expression of the TCA cycle or FAO markers, thus excluding a direct effect of MR blockade on fat metabolism in SM (Figure 5B, genes listed in Table 1). Moreover, given the co-occurrence of IR and mitochondrial dysfunction in SM, we evaluated the protein expression of the transcriptional regulator PGC-1α in GA in order to investigate the potential implication of MR in SM mitochondrial biogenesis. As expected, mice fed an HFD showed a significant reduction in PGC-1α protein levels, as well as in *mtTFA* and *cytochrome c* mRNA expression in GA. The MR blockade did not affect the mentioned mitochondrial biogenesis marker expression in the HFD + Spiro group, suggesting that Spiro was not able to improve mitochondrial function in SM of obese mice (Figure S1, listed in Table 1). In addition, analysis of *Col3a1* gene expression suggested that pharmacological MR blockade did not influence ECM remodeling in the SM of mice challenged with HFD (Figure 5B).

Finally, Chadwick and colleagues demonstrated that MR antagonism by Spiro was able to downregulate the *Ankrd1* transcript, a sarcomeric component that is altered in several muscle diseases [55], as observed in a Duchenne muscular dystrophy (DMD) mouse model, thus identifying this adaptor protein as a novel MR gene target [22]. By contrast, in our model, Spiro did not induce significant changes in Ankrd1 gene expression, despite a positive trend in terms of increased mRNA levels that were observed in the HFD + Spiro group compared with HFD-fed mice (Figure 5B). On the other hand, the effect of MR antagonism on myokine profiles needs to be clarified. Taken together, our data indicate that reduced activity of SM MR does not improve insulin signaling in mouse myocytes, excluding any protective effect of SM MR pharmacological blockade upon local and systemic insulin sensitivity. However, the physiological role of MR in SM is still debated, and further studies are required to elucidate the mechanisms underlying the effects of MR antagonism in this tissue.

## 4. Materials and Methods

### 4.1. Animal Model

Animal procedures were approved by the Italian National Institutes of Health Care and Use Committees (approval number 493/2016-PR). Male 10-week-old C57BL/6J mice (Charles River Laboratories, Calco, Italy) were fed either a normal diet (ND; 10% kcal as fat; D12450B; Research Diets, New Brunswick, NJ, USA), a high-fat diet (HFD) (60% kcal as fat; D12492; Research Diets), or an HFD containing Spironolactone (0.165 gr/Kg of diet) for 12 weeks. Mice were divided into 3 groups (n = 10) as follows: mice fed an ND (ND group); mice fed an HFD (HFD group); and mice fed an HFD plus Spiro (HFD + Spiro group). All in vivo tests were performed at the end of the treatment.

### 4.2. Glucose Tolerance Assessment

Animals were denied access to food for 6 h, and a sample of blood was collected from the tail to measure basal blood glucose levels. The animals were then injected with a glucose solution into the peritoneum (0.09 g/mL glucose and 112 µL solution/10 g body weight), and blood glucose concentrations were measured using a commercial glucometer (FreestyleFreedom Lite; Abbott, Alameda, CA, USA) over the next 2 h at 20-min intervals for the first hour and at 30-min intervals during the second hour.

### 4.3. Gene Expression Analysis

Total RNA was isolated from snap-frozen gastrocnemius using the RNeasy tissue mini kit (Qiagen, Milan, Italy) following the manufacturer’s instructions. The purity, integrity, and yield of RNA were analyzed by Agilent Technologies 2001 bioanalyzer using the RNA 6000 LabChip kit. Total RNA (1 µg) was treated with RNase-Free DNase 1 (Qiagen) and reverse transcribed using the High-Capacity cDNA Reverse Transcription System (Applied Biosystems (Thermo Fisher Scientific), Milan, Italy) according to the manufacturer’s instructions. qRT-PCR assays were performed in 96-well optical plates using Mx3000P LightCycler instrument (Stratagene, Milan, Italy). Each cDNA sample was analyzed in duplicates using gene-specific primers spanning intron/exon boundaries for gene expression quantification (see Table 1 for primer sequences) and Fast SYBR Green Master Mix (Applied Biosystems (Thermo Fisher Scientific), Milan, Italy). The following reaction mixture per well was used: 5 µL of Fast Syber Green 2x (Applied Biosystems), 1 µL of primer mix at a final concentration of 250 nM, 2 µL of RNase-free water, and 2 µL of cDNA. Quantitative normalization of cDNA in each sample was performed using either 18S ribosomal RNA or TATA-box binding protein (Tbp) as an internal control. Relative quantification was calculated using the 2^−∆∆CT^ method.

### 4.4. Histological Analysis

Tibialis anterior samples from all study groups were fixed in 4% paraformaldehyde solution, pH 6.9 (EMD Millipore Corporation, Billerica, MA, USA), and were embedded progressively with increasing concentrations of sucrose. They were subsequently embedded in Tissue-Tek OCT compound to be snap-frozen in 2-methyl-butane and immersed in liquid nitrogen as previously described [56]. Each sample was cut (7 µm thick), and the cutting plane corresponded to the largest surface used for histological examination and morphometry evaluation by hematoxylin-eosin (H&E) staining.

Prepared slides were stained using the Oil Red O method for fat deposition, and six fields at 10 and 20-fold magnification for each slide were analyzed. The images were subsequently analyzed using ImageJ, an open-source software.

### 4.5. Cell Culture and Treatments

Mouse C2C12 myoblasts (American Type Culture Collection) were maintained in DMEM at pH 7.4, containing 4.5 mg/mL glucose, 10% FCS, and penicillin/streptomycin (500 U/mL each), with 95% O2/5% CO2, at 37 °C. For differentiation, myoblasts were seeded in 6-well plates (60,000 cells per well) and cultivated in the same medium until 90% confluence. After this period, cells were differentiated in DMEM containing 4.5 mg/mL glucose, 2% horse serum, and penicillin/streptomycin (500 U/mL each) until the formation of myotubes (5 days). Medium was changed every 2 days.

Palmitate was prepared as described previously [57]. Briefly, palmitate was dissolved in 0.1 M NaOH by heating at 70 °C. After filtration, the solution was then diluted with 10% fatty acid-free BSA and stored at −20 °C. Palmitate treatment was performed with the concentrations indicated in the figure legends for generally 16 h. In all experiments, 2 h before the treatment, the culture medium was changed to serum-free DMEM. For protein phosphorylation detection, 100 nM insulin (Sigma Aldrich, Milan, Italy) was added for 15 min before cell lysates were harvested. Spiro was dissolved in ethanol and added to the culture medium at a final concentration of 1 µM together with palmitate.

### 4.6. Glucose Uptake Analysis

The amount of glucose uptake in C2C12 myotubes was measured using the Glucose Uptake-Glo Assay Kit (Promega, Madison, WI, USA) according to the manufacturer’s instructions. For measuring glucose uptake in C2C12 cells based on the detection of 2-deoxyglucose-6-phosphate, after 16 h of treatment, C2C12 myotubes were placed in 1 mL of glucose-free medium containing 2-deoxyglucose-6-phosphate in 96-well plates for 3 h. The luminescence intensity in the cells was then analyzed with a luminometer.

### 4.7. Western Blot Analysis

Gastrocnemius (n = 6) or C2C12 cell pellets were lysed at 4 °C in HNTG lysis buffer (1% Triton X-100, 50 mM HEPES, 10% glycerol, 150 mM NaCl, 1% sodium deoxycholate) supplemented with phosphatase inhibitor cocktail 2 and 3 (Sigma Aldrich, Milan, Italy) and protease inhibitor cocktail (Sigma Aldrich, Milan, Italy). A clear supernatant was obtained by centrifugation of lysates at 13,000× *g* for 15 min at 4 °C. Protein concentration was determined by a BCA protein assay kit (Pierce; Thermo Fisher Scientific, Milan, Italy). Protein samples were subjected to sodium dodecyl sulfate polyacrylamide gel electrophoresis (SDS-PAGE) using Miniprotean precast gels (BioRad; Segrate, Italy) and electroblotted onto nitrocellulose membranes (Bio-Rad, Segrate, Italy). Membranes were blocked for 1 h at room temperature (RT) with 5% non-fat milk in Tris-buffered saline with 0.05% Tween 20 (TBS-T). Incubation with primary specific antibodies was performed in a blocking solution (5% milk or bovine serum albumin in TBS-T) overnight at 4 °C and with horseradish peroxidase-conjugated secondary antibodies (in blocking solution) for 1 h at RT. We used antibodies against Akt 1:1000 (9272; Cell Signaling, Danvers, MA, USA), phosphor-Akt (Ser473) 1:1000 (9271; Cell Signaling, Danvers, MA), and PGC-1α 1:1000 (AB3242; Millipore). 1D5 MR antibody, kindly provided by dr Gomez-Sanchez [58].

The appropriate secondary horseradish peroxidase-conjugated antibodies from Jackson Immunoresearch were used in the blocking solution (1:5000). Immunoreactive bands were visualized by Luminata Forte Western chemiluminescent HRP substrate (Millipore (Merk); Milan, Italy) using ImageQuant LAS 4000 (GE Healthcare). Equal sample loading was confirmed by α-Tubulin 1:1000 (T5168, Sigma Aldrich, Milan, Italy), and bands were quantified by densitometry using the ImageQuant TL software from GE Healthcare Life Sciences.

### 4.8. Statistical Analysis

Data are reported as means ± standard error of the mean (SEM). Data points > ±2 standard deviations (SD) from the mean were considered statistical outliers and were excluded from all analyses (ROUT method). Statistical comparisons were made by 1- or 2-way ANOVA tests, followed by Bonferroni multiple comparison post hoc analysis or a student’s *t*-test, using Prism 8.0 (GraphPad, San Diego, CA, USA). Values of *p* < 0.05 were considered significant.

## 5. Conclusions

A number of studies have shown that, at least in murine models of obesity, pharmacological MR blockade improves glucose metabolism. MR antagonism promotes BAT and stimulates the “browning” of white adipose tissue, which represents processes that may protect glucose homeostasis [20,27]. Our findings exclude the possibility that MR blockade in SM contributes to counteracting the glycemic disorders in obese mice, which are improved by the administration of MRA. Interestingly, MR antagonism modulates the expression of the myokine irisin, which has been shown to stimulate brown fat thermogenesis [52], potentially leading to beneficial metabolic effects, thus suggesting MRA treatment as a valid pharmacological approach in clinical programs of cardiometabolic rehabilitation.

## Figures and Tables

**Table 1:** Sequences of primers used in qRT-PCR.

	Primers, 5′-3′	
Gene	Forward	Reverse
*Interleukin-6 (IL-6)*	GGAGAGGAGACTTCACAGAGG	CCAGTTTGGTAGCATCCATC
*Ankyrin repeat domain 1 (ANKRD1)*	AGACTCCTTCAGCCAACATGATG	CTCTCCATCTCTGAAATCCTCAGG
*Fndc5 (Irisin)*	CAACGAGCCCAATAACAACA	AGAAGGTCCTCTCGCATTCTC
*aconitase 2 (Aco2)*	TCTCTAACAACCTGCTCATCGG	TCATCTCCAATCACCACCCACC
*acyl-Coenzyme A dehydrogenase, C-4 to C-12 straight chain (Acadm)*	AGTACCCTGTGGAGAAGCTGAT	TCAATGTGCTCACGAGCTATG
*Isocitrate dehydrogenase subunit alpha (Idh3a)*	AGGACTGATTGGAGGTCTTGG	ATCACAGCACTAAGCAGGAGG
*Malate dehydrogenase 2 (Mdh2)*	GACCTGTTCAACACCAACGC	GGATGGTGGAGTTCACTGGG
*Succinate dehydrogenase complex subunit C (Sdh-c)*	GAAGAAGAACACGAGTTCAAACC	GTGCCATAGGAAGAGACCATTT
*Succinate dehydrogenase cytochrome b small subunit (Sdh-d)*	CCTGCTCTGTGGTGGACTACT	CCCATGAACGTAGTCGGTAAC
*TATA box binding protein (TBP)*	GGACCAGAACAACAGCCTTC	CCGTAAGGCATCATTGGACT
*NR3C2, nuclear receptor subfamily 3, group C, member 2 (MR)*	CTGGTTCCTCAGCTCTCCAC	GGATCATCTGTTTGCCTGCT
*RN18S1, 18S ribosomal RNA (18S)*	CGGCTACCACATCCAAGGAA	GCTGGAATTACCGCGGCT
*Mitochondrial transcription factor A (mtTFA)*	CCG AGG TGG TTT TCA TCT GT	GCT GAA CGA GGT CTT TTT GG
*Cytochrome C (Cyt C)*	GAGGCAAGCATAAGACTGGA	TACTCCATCAGGGTATCCTC
*Col3a1*	CCT CTG GTT CTC CTG GTC TG	CCA CCT TCA CCC TTA TCT CC
*Monocyte chemoattractant protein-1 (MCP-1)*	CCCAATGAGTAGGCTGGAGA	TTCTGGACCCATTCCTTCTTG

## Data Availability

All materials, data, and protocols associated with this publication will be made available to readers upon request.

## Supplementary Materials

The supporting information can be downloaded at: https://www.mdpi.com/article/10.3390/ijms24087412/s1.
