# Supplementary material for: Neutral Effect of Skeletal Muscle Mineralocorticoid Receptor on Glucose Metabolism in Mice
Source: Int J Mol Sci. 2023 Apr 18;24(8):7412. doi: 10.3390/ijms24087412 (PMC10139152; doi:10.3390/ijms24087412)
Supplement: Supplementary file 1 [file ijms-24-07412-s001.zip › ijms-2289352-supplementary.pdf]

Supplementary Materials

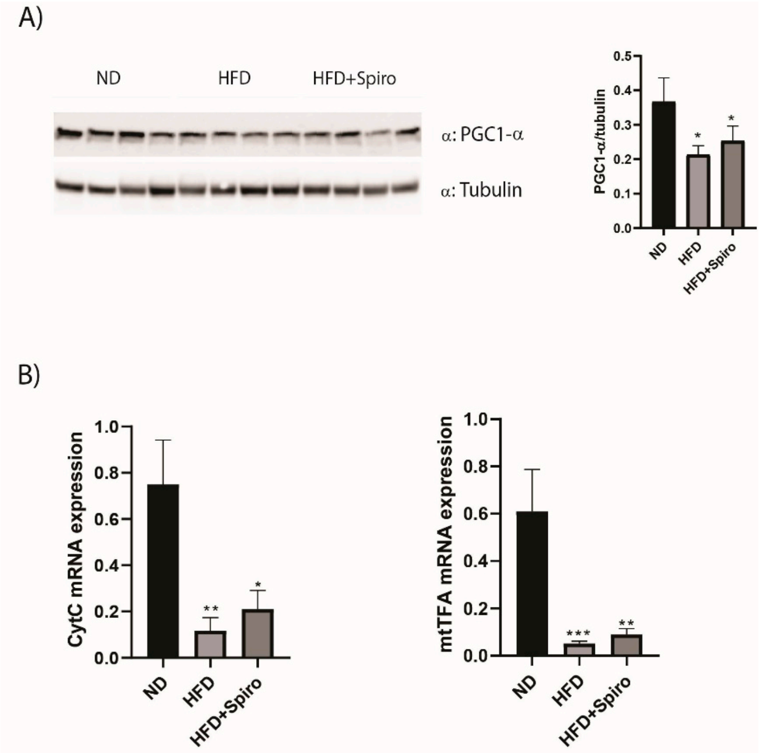

Figure S1. Mice fed a HFD showed mitochondrial dysfunction in SM, but Spironolactone treatment did not this effect. A, representative immunoblots of PGC1-α analysis in GA (n=10) and distribution graphs of the densitometric scanning analyses performed by ImageQuant TL software by using α-tubulin as loading control. B, qRT-PCR analysis, of genes related to mtTFA and cytochrome c in GA of ND, HFD and HFD + Spiro groups (n=10). Values are expressed as means ± SEM.*P < 0.05, vs ND, **P < 0.005, vs ND, ***P< 0,001 vs ND.
